# Physical activity: the key to life satisfaction - correlations between physical activity, sedentary lifestyle, and life satisfaction among young adults before and after the COVID-19 pandemic

**DOI:** 10.3389/fpubh.2025.1486785

**Published:** 2025-02-17

**Authors:** Éva Bácsné Bába, Attila Lengyel, Christa Pfau, Anetta Müller, Éva Judit Bartha, Renátó Balogh, György Szabados, Zoltán Bács, Gergely Ráthonyi

**Affiliations:** ^1^Faculty of Economics and Business, Institute of Sport Economics and Management, University of Debrecen, Debrecen, Hungary; ^2^Faculty of Economics and Business, Coordination Center for Research in Social Sciences, University of Debrecen, Debrecen, Hungary; ^3^Faculty of Economics and Business, Institute of Accounting and Finance, University of Debrecen, Debrecen, Hungary

**Keywords:** sitting, physical activity, university students, SBQ, IPAQ, life satisfaction

## Abstract

COVID-19 has fundamentally altered the world, significantly affecting people’s health and quality of life. These changes may have impacted young adults’ physical activity (PA) and sedentary behavior (SB). This study aims to explore lifestyle changes among young adults, particularly focusing on the relationship between physical activity, sedentary behavior, and life satisfaction before and after the COVID-19 pandemic. To achieve this, we analyzed two surveys carried out in 2018 and 2024. A total of 799 participants were recruited for this age group, with 279 in 2018 and 520 in 2024. We employed *T*-tests, Pearson’s Chi-square tests, Mann–Whitney tests, and ordinal linear regression to uncover associations between physical activity and sitting time across the two periods, alongside various socio-demographic factors and predictors of life satisfaction. Our results reveal notable trends and changes in physical activity levels and sitting times among young adults in Hungary between 2018 and 2024. The most striking changes occurred in IPAQ (International Physical Activity Questionnaire) categorical scores, indicating a shift in activity levels across several demographic groups. The level of physical activity increased for both male and female respondents, residents of county seats and cities, as well as those living in relationships. In 2018, young Hungarian adults who maintained a balance of physical activity—including cycling, some home-based physical work, and limited sitting time—tended to report higher life satisfaction. In 2024, an interesting pattern emerged regarding leisure activities: individuals who engaged in very hard exercise during their free time reported varying levels of life satisfaction. Those who never engaged in very hard exercise or did so only once a week reported lower satisfaction compared to those who exercised daily. This finding aligns with existing research on the mental health benefits of regular intense physical activity. Furthermore, individuals whose physical activity was primarily work-related reported significantly lower life satisfaction compared to those whose physical activity was primarily leisure-related. This suggests that the context of physical activity is crucial in determining its impact on life satisfaction.

## 1 Introduction

Regular physical activity not only protects against chronic diseases but also improves the quality of life (1–3) and has a positive impact on subjective well-being across all age groups (4–9). Additionally, it ensures healthy growth in young people, enhances thinking, learning, and judgment (10). According to the World Health Organization’s global status report, globally, 1 in 4 adults do not meet the recommended levels of physical activity. More than 80% of the world’s adolescent population is insufficiently physically active. Inactive individuals have a 20–30% higher mortality risk compared to adequately active populations (10).

Since 2002, the Eurobarometer surveys on sport and physical activity have been conducted quadrennially to examine EU citizens’ physical activity patterns, including frequency, location, and motivations. They explore factors that encourage or hinder active lifestyles, such as health benefits, social experiences, lack of time, or motivation. The surveys highlight regional differences, with Northern Europeans generally being more active compared to Southern and Eastern Europeans. These findings provide valuable insights for EU policymakers to develop programs promoting active living. They indicate that among the 15–24 age group, the proportion of those who never or rarely engage in regular exercise shows a declining trend (11–13). According to the 2022 survey, across European Union member states, 27% of young men and 46% of young women do not engage in regular weekly exercise. In Hungary, 35% of young men and 46% of young women lead a sedentary lifestyle. Young people in neighboring countries are generally more active than Hungarians, except in Romania, where 57% of men and 51% of women never or rarely exercise (13). In the Czech Republic, the situation is reversed, with 15-24-year-old women being less inactive than their male counterparts. “Age decreases physical activity, while higher education and financial security increase it (13). Research shows a significant decline in physical activity during the transition from high school to university, with one study finding that while 66.2% of students met recommended activity levels in high school, only 44.1% maintained these levels during their first weeks at university (14). Students face various adjustment challenges during this transition period (15), with physical activity levels particularly affected during exam periods (16). Compared to the general young adult population, university students spend more time sitting, with this time increasing over the past decade (17). Research conducted in Germany found that first-year students are significantly less active than upper-year students (18–20). In Edelmann et al.’s (19) study, approximately 22.4% of the participating students did not meet WHO’s physical activity recommendations. Additionally, 47.6% of the students sat for 8 h or more daily.”

The transition from high school to university is characterized by lifestyle changes that often lead to increased risk behaviors (21). Gender-wise, young men are more active compared to women (22) and women engage in less intensive activities than their male counterparts (23). Several studies have found that young people who engage in higher physical activity levels also tend to have high sedentary time (24–26). Compared to the general young adult population, university students spend more time sitting, with this time increasing over the past decade (17). The spread of a sedentary lifestyle is significantly influenced by the continuous development of new technologies aimed at making daily life easier and work more efficient, many of which have now become sedentary activities (27). Over the past decades, the nature of human work has undergone a significant transformation. Many “classical” jobs, typically requiring physical labor (such as in agriculture or manufacturing), have disappeared due to robotization (28). Additionally, with the spread of digitalization, desk jobs have emerged (29).

The DESI (Digital Economy and Society Index) is an annual indicator published by the European Union that measures the digital development and competitiveness of EU member states. It evaluates four main areas: connectivity (internet access and network infrastructure), digital skills, internet usage, and the integration of digital technologies into businesses and the digitalization of public services. The index aims to promote digital transformation and reduce disparities between countries. DESI has shown interesting correlations with lifestyle changes. According to Chareonwongsak (30), household tasks have become simpler with the help of new technological devices, and motorized vehicles have made commuting to work easier and more convenient.

Brey (31) argues that digitalization has generally improved our quality of life, although its negative effects should not be overlooked. Sitting during work, driving, or using public transport, watching TV, or even playing board games (32) falls under sedentary behavior (SB) if done in a sitting or lying position. In developed countries, the time spent sitting has continuously increased over the past decades. In developed countries, sitting time represents a significant portion of daily activity. More than a decade ago, Bauman et al. (33), using accelerometer data, found that sitting time accounted for 55–70% of adults’ waking hours, with average sitting work time being ≥9 h per day. Moreno-Lamas et al. (28) found a correlation between digital development and sedentary lifestyle in their study. They used the 2018 Eurobarometer data and the Digital Economy and Society Index (DESI) employed by the European Commission. Their analysis showed that higher DESI scores were associated with increased sitting time. They also found that ownership of electronic devices (desktop computers, laptops, DVD/CD players) and internet connectivity was significantly associated with extended sitting times (>4.5 h per day). Barkley et al. (34) revealed a connection between mobile phone usage and a sedentary lifestyle, concluding that heavy mobile phone users (495.1 ± 227.6 min/day) had significantly (18–25%) higher daily sitting times. According to Pandaya et al. (35), COVID-19 has significantly increased the use of digital devices. The use of digital devices increased to an average of 5 h per day, with some heavy users reaching up to 17.5 h of screen time per day (36). A survey conducted in the United States found that weekly leisure screen time increased from 25.9 ± 11.9 h in 2018 to 28.5 ± 11.6 h during the pandemic, largely driven by TV shows and various streaming services (37). The use of digital devices is particularly characteristic of young people, and previous research has identified this as a potential risk factor for physical inactivity in this group.

The restrictions introduced worldwide in 2020 due to the COVID-19 pandemic have significantly contributed to the increase in sedentary lifestyles. Numerous studies have confirmed the substantial decrease in physical activity among various national populations due to the pandemic (38–45). Sanudo et al. (46) used the IPAQ to assess changes in physical activity among Spanish youth compared to the period before the lockdowns. Their results also indicate a significant decline. While 84% of respondents met the WHO’s physical activity guidelines before the lockdowns—which recommend 150 min of moderate physical activity per week, 75 min of vigorous physical activity per week, or an equivalent combination of moderate and vigorous intensity physical activity—this figure fell to 74% during COVID. Subjectively measured walking time (minutes/day), the amount of moderate and vigorous intensity physical activities significantly decreased during the lockdowns, while subjectively measured sitting time significantly increased during this period. The lifestyle changes that emerged during this period remain a subject of research (47).

Changes resulting from the pandemic or innovation have a significant impact on people’s quality of life. Besides physical health, a crucial factor in our lives is a positive quality of life (QoL), i.e., positive mental health, or well-being. One of the most frequently studied components of well-being is life satisfaction. According to Cheng (48), life satisfaction indicates how much an individual loves the life they live. In this sense, “life” can be defined as all areas of an individual’s life at a given moment, as well as the individual’s overall assessment of their life. Fujita and Diener (49) suggest that an individual’s life satisfaction remains relatively stable, apart from minor positive or negative fluctuations. Life satisfaction data reflect the quality of life characteristic of a country or a specific social group. This allows for the assessment of the scale of a social problem and the formulation of intervention opportunities for policymakers. High life satisfaction indicates a good quality of life for the population, while low satisfaction suggests the existence of some problem (50). The examination of subjective well-being first appeared in official statistics in 2013, in the SILC survey coordinated by Eurostat and carried out with a uniform methodology in 32 countries. The questionnaire used measured respondents’ satisfaction with life and important areas of life through 22 questions. In Hungary, nearly 18,000 individuals aged 16 and older responded to the subjective well-being questionnaire. The variable measuring life satisfaction is the most frequently cited indicator from the examination of subjective well-being. Respondents rated their overall life satisfaction on an 11-point scale ranging from 0 to 10 (0 = not satisfied at all, 10 = very satisfied). Among countries, there are no significant differences except for the lowest indicators found in Bulgaria and Serbia. The average score for the entire European Union was 7.1 points in 2013. Denmark, Finland, Sweden, and Switzerland topped the ranking with an average score of 8.0 points. Hungary’s average score was 6.2 points, placing it in a tie with Cyprus, Greece, and Portugal, ahead of Bulgaria and Serbia. A north–south divide in life satisfaction is observed, with the highest satisfaction in Scandinavian countries and generally lower satisfaction in Southern European countries. There are significant differences between countries when broken down by age groups. In Hungary, life satisfaction markedly decreases with age. The possession or lack of material goods affects individuals’ subjective quality of life. Material wealth can be considered a probabilistic condition for subjective well-being. This is supported by life satisfaction results examined along income lines (51).

Given this context and the unique challenges faced by young adults in Hungary, our study specifically focuses on individuals aged 18–30, examining their physical activity patterns, sedentary behavior, and life satisfaction. The age range was chosen to capture the critical period of transition from education to work life, and the comparison between 2018 and 2024 allows us to assess both pre- and post-pandemic changes in these patterns.

## 2 Methods

### 2.1 Study sample

The sampling strategies and data collection methods for this study differed between the two time points, which affects comparability and requires careful interpretation. In 2018, a stratified random sampling approach was employed through a mail survey conducted by Synapsis, a market research company in Debrecen, Hungary. The initial sample (*N* = 1,343) was nationally representative of the Hungarian adult population. For this study, we extracted data for young adults aged 18–30 (*N* = 276).

In 2024, data were collected using a convenience sampling method through community websites, specifically targeting young adults aged 18–30 (*N* = 520). To ensure comparability with the 2018 random sample, post-stratification weights were applied to adjust for demographic differences in gender, settlement type, and education between the two samples. Despite lower response rates of 48% in 2018 and 42% in 2024, non-response bias analyses showed no significant demographic deviations within either sample. These statistical adjustments enable meaningful comparisons between the two time points.

In 2018, 48.8% of respondents were male and 51.2% were female. In 2024, males accounted for 51.5% and females for 48.5%. Settlement type showed notable variation. In 2018, 15.0% of respondents resided in the capital city, compared to only 2.4% in 2024. At the same time, residence in cities increased from 36.7 to 49.7%, while county towns accounted for 27.5% in 2018 and 25.5% in 2024. The proportion living in municipalities rose from 20.8 to 22.5%.

In the 2018 sample 64.6% of respondents were single, 16.9% were in a relationship, and 16.8% were married. In the 2024 sample, 52.9% were single, 45.5% were in a relationship, while only 1.6% were married. Economic status highlights the substantial proportion of students in 2024. In 2018, 52.2% were in full-time employment, while 24.0% were students. In contrast, by 2024, only 6.1% reported full-time employment, while 75.9% identified as full-time students. Family income patterns suggest improved economic conditions for those reporting values.

In 2018, 22.5% of respondents earned less than 200,000 HUF monthly, compared to just 2.6% in 2024. The proportion earning more than 550,000 HUF increased from 8.9 to 22.7%, although 41.0% of respondents in 2024 chose not to disclose their income, up from 10.9% in 2018. Education levels also displayed notable differences.

In 2018, 25.9% of respondents had completed high school, increasing to 69.3% in 2024. Conversely, the proportion with bachelor’s degrees fell from 16.6 to 2.7%, while those with master’s degrees declined from 17.5 to 0.7%. These differences primarily reflect the recruitment of a younger, more education-focused sample in 2024 through community websites, in contrast to the broader stratified sample in 2018.

The study collected data on physical activity, sedentary behavior, mental health, and sports habits among Hungarian adults using well-established and validated questionnaires. In a previous publication, we presented findings on the physical activity levels (52) and sedentary behavior (53) of the Hungarian adult population. However, this manuscript focuses specifically on the changes in physical activity and sedentary behavior among young adults (aged 18 to 30 years) before and after the COVID-19 pandemic, with special attention to their life satisfaction.

### 2.2 Instrument

Physical activity levels were assessed using the long form of the International Physical Activity Questionnaire (IPAQ). To evaluate sedentary behavior, we utilized the Sedentary Behavior Questionnaire (SBQ). Both instruments have been validated in multiple languages and cultural contexts, though they carry inherent limitations common to self-reported measures. To minimize recall bias, participants were provided with detailed instructions and examples for activity classification, and responses were screened for logical inconsistencies. The IPAQ is structured to measure physical activity across various domains, including work, transportation, household tasks, leisure, and time spent sitting, over the past 7 days. The instrument’s 7-day recall period was chosen to balance accuracy of recall with representation of typical behavior patterns. The IPAQ scoring protocol was applied to interpret the data, with physical activity responses being converted into metabolic equivalent tasks (METs). Based on MET values, physical activity was categorized into three levels: low, moderate, and high. These categories are referred to in the text as IPAQ scores.7.

The SBQ was adapted from Rosenberg et al. (54). The questionnaire demonstrates good test–retest reliability (ICC = 0.85) and criterion validity against accelerometer data (*r* = 0.71). The primary goal of this questionnaire is to assess sedentary time across nine different activities, including watching television, playing computer or video games, sitting while listening to music, talking on the phone, doing paperwork or office tasks, sitting and reading, playing a musical instrument, engaging in arts and crafts, and sitting while driving or traveling by car, bus, or train, for both weekdays and weekends. The SBQ scoring protocol was utilized for data analysis. In our version of the questionnaire, we incorporated additional details regarding modern info-communication devices. The online self-reported questionnaire also collected data on participants’ personal and socio-demographic characteristics, such as gender, age, type of residence, region, marital status, education, occupation, household income, and life satisfaction. To validate response quality, the online platform included automated checks for completeness and logical consistency.

### 2.3 Statistical analysis

The data analysis was conducted using the Statistical Package for Social Sciences (SPSS Inc., Version 28.0, Chicago, IL, USA). Statistical significance was defined as *p* < 0.05 for all tests. For transparency and reproducibility, we report effect sizes, confidence intervals, and exact *p*-values throughout our results. The choice of ordinal regression for analyzing life satisfaction was based on the ordinal nature of the dependent variable (11-point scale) and the mix of categorical and continuous predictors. Prior to analysis, we tested key assumptions including the absence of multicollinearity (all VIF < 2.5) and proportional odds (Test of Parallel Lines, *p* > 0.05). Potential confounders were identified through literature review and preliminary analyses, with age, gender, and education level included as control variables in the final models. Pearson’s Chi-square and Cramer’s V tests were employed to examine differences between the two periods concerning the physical activity levels of participants across various socio-demographic characteristics. In order to reveal the factors that predicts life satisfaction, regression analyses, ordinal regression models were used. The exclusion of certain variables, such as detailed socioeconomic status measures, was based on preliminary analyses showing high correlation with included variables (*r* > 0.7) and potential multicollinearity issues. The change in socio-demographics and life satisfaction between the 2018 and 2024 samples of young Hungarian adults were measured by Mann–Whitney *U* tests. Independent samples *t*-tests were conducted to compare leisure time activities on weekends between the 2018 and 2024 samples. Although the Shapiro-Wilks and the Levene tests proved significant (*p* ≤ 0.05) for some of the variables, we decided to employ the independent samples *T*-test for investigate the change in physical activity levels. According to the central limit theorem, with large sample sizes (>30) the sampling distribution tends to follow a normal distribution regardless of the shape of the underlying population distribution. Therefore, even with non-normal data, the *t*-test on the sample means can be assumed to follow a normal distribution. Furthermore, with large sample sizes, the impact of variance heterogeneity is reduced, as sample means provide reliable estimates of population means. The *t*-test is robust to this violation with balanced sample sizes exceeding approximately 200, as is the case in this study.

## 3 Results

### 3.1 IPAQ and SBQ results

When interpreting demographic differences and trends in physical activity, sedentary behavior, and life satisfaction between 2018 and 2024, it is important to consider both real changes over time and the methodological differences in sampling (Table 1).

**Table 1:** IPAQ and SBQ differencees among young adults between 2018 and 2024.

Variable	2018 IPAQ *N* (%)			2018 sitting time (Mean, 95% CI)		2024 IPAQ *N* (%)			2024 sitting time (Mean, 95% CI)		Statistical tests
	Low	Mod	High	Weekday	Weekend	Low	Mod	High	Weekday	Weekend	IPAQ
Gender											
Male	15 (11.11)	28 (20.74)	92 (68.15)	335.82 (320.4–351.2)	270.4 (256.8–284.0)	29 (10.86)	33 (12.36)	205 (76.78)	271.2 (258.9–283.5)	243.88 (232.1–255.7)	*χ*^2^: *p* = 0.800; *V* = 0.023
Female	25 (17.61)	47 (33.09)	70 (49.30)	294.15 (279.8–308.5)	242.27 (229.4–255.1)	48 (19.05)	42 (16.67)	162 (64.28)	342.23 (328.9–355.6)	275.63 (263.8–287.5)	*χ*^2^: *p* = 0.001; *V* = 0.191
Settlement											
Capital city	8 (19.05)	16 (38.10)	18 (42.85)	382.38 (361.3–403.5)	319.45 (299.8–339.1)	1 (8.33)	1 (8.33)	10 (83.34)	425.45 (398.2–452.7)	223.96 (203.1–244.8)	*χ*^2^: *p* = 0.45; *V* = 0.339
County seat	12 (15.79)	23 (30.26)	41 (53.95)	316.21 (298.9–333.5)	257.05 (241.6–272.5)	17 (12.88)	16 (12.12)	99 (75.00)	296.65 (281.2–312.1)	223.35 (209.5–237.2)	*χ*^2^: *p* = 0.003; *V* = 0.240
City	10 (9.80)	23 (22.55)	69 (67.65)	298.56 (282.6–314.5)	259.94 (245.9–273.9)	50 (19.38)	37 (14.34)	171 (66.28)	301.28 (287.9–314.7)	263.26 (251.4–275.1)	*χ*^2^: *p* = 0.030; *V* = 0.139
Municipality	9 (15.52)	14 (24.14)	35 (60.34)	292.36 (274.8–309.9)	203.12 (189.3–216.9)	8 (6.90)	21 (18.10)	87 (75.00)	313.62 (297.9–329.3)	292.97 (279.3–306.6)	*χ*^2^: *p* = 0.90
Employment status											
Employed	24 (13.48)	41 (23.03)	113 (63.49)	316.31 (301.5–331.1)	251.55 (239.0–264.1)	7 (9.33)	2 (2.67)	66 (88.00)	273.7 (257.3–290.1)	296.45 (281.6–311.3)	*χ*^2^: *p* = 0.000; *V* = 0.267
Unemployed	15 (15.31)	35 (35.71)	48 (48.98)	310.63 (293.0–328.3)	263.01 (247.2–278.8)	71 (15.95)	73 (16.40)	301 (67.65)	311.48 (299.0–323.9)	252.54 (242.4–262.7)	*χ*^2^: *p* = 0.000; *V* = 0.189
Marital status											
Relationship	30 (16.39)	43 (23.50)	110 (60.11)	324.77 (309.5–340.0)	271.89 (258.3–285.5)	39 (14.18)	41 (14.91)	195 (70.91)	321.5 (307.6–335.4)	271.57 (259.7–283.4)	*χ*^2^: *p* = 0.035; *V* = 0.121
No relationship	9 (9.68)	32 (34.41)	52 (55.91)	294.24 (277.6–310.9)	224.84 (210.3–239.4)	38 (15.51)	35 (14.29)	172 (70.20)	288.25 (274.8–301.7)	244.6 (233.2–256.0)	*χ*^2^: *p* = 0.000; *V* = 0.228

Statistical analyses revealed several significant patterns in physical activity levels and sedentary behavior between 2018 and 2024, with some findings showing unexpected trends that warrant careful interpretation. In 2018, 11.11% of male respondents had low, 20.74% moderate and 68.15% high levels of physical activity. Their mean sitting times were 335.82 min on weekdays and 270.4 min on weekends. In 2024, 10.86% of male respondents had low, 12.36% moderate and 76.78% high levels of physical activity, with mean sitting times of 271.2 min on weekdays and 243.88 min on weekends. The Chi-square test showed no significant difference in IPAQ scores between the years (*p* = 0.800), but the *t*-test indicated a significant reduction in weekday sitting time (*p* = 0.007, Md = 64.622).

In 2018, 17.61% of female respondents had low, 33.09% moderate and 49.30% high levels of physical activity, with sitting times of 294.15 min on weekdays and 242.27 min on weekends. In 2024, 19.05% of female had low, 16.67% moderate, and 64.28% high levels of physical activity, with sitting times of 342.23 min on weekdays and 275.63 min on weekends. The Chi-square test indicated significant differences in IPAQ scores (*p* = 0.001, *v* = 0.191), but no significant change in sitting time (*p* = 0.071 for weekdays, *p* = 0.225 for weekends). Settlement patterns revealed varying trends across different urban contexts. Respondents from the capital city showed no significant change in IPAQ scores (*p* = 0.45, *v* = 0.339), but there was a significant change in weekend sitting time (*p* = 0.112). County seat respondents showed significant changes in IPAQ scores (*p* = 0.003, *v* = 0.240) but no significant changes in sitting times. City respondents also showed significant differences in IPAQ scores (*p* = 0.030, *v* = 0.139), but no significant changes in sitting times. Municipality respondents did not show significant changes in IPAQ scores (*p* = 0.90), but there was a significant change in weekend sitting time (*p* = 0.028, Md = −89.85).

Employment status emerged as a significant factor in activity patterns. Employed individuals showed a significant change in IPAQ scores (*p* = 0.000, *v* = 0.267), but no significant change in sitting times. Unemployed individuals also showed significant changes in IPAQ scores (*p* = 0.000, *v* = 0.189), but no significant changes in sitting times.

Work-type analysis revealed particularly interesting patterns, especially regarding standing jobs. Respondents with sitting activities showed significant changes in IPAQ scores (*p* = 0.000, *v* = 0.240) and a significant reduction in weekday sitting time (*p* = 0.010, Md = 60.302). Those with mixed activities did not show significant changes in IPAQ scores (*p* = 0.696) or sitting times. Standing activity respondents showed marginal significance in IPAQ scores (*p* = 0.072), but no significant changes in sitting times. This finding about standing jobs warrants particular attention as it suggests potential occupational health implications. Physical activity respondents did not show significant changes in IPAQ scores (*p* = 0.888), but there was a significant change in weekday sitting time (*p* = 0.028, Md = −81.714).

Relationship status analysis revealed that both groups showed changes in physical activity levels. Individuals in relationships showed significant changes in IPAQ scores (*p* = 0.035, *v* = 0.121), but no significant changes in sitting times. Similarly, those not in relationships showed significant changes in IPAQ scores (*p* = 0.000, *v* = 0.228), but no significant changes in sitting times.

Respondents with primary education showed no significant changes in IPAQ scores (*p* = 0.319). Those with secondary education showed significant changes in IPAQ scores (*p* = 0.014, *v* = 0.117), but no significant changes in sitting times. University-educated respondents showed significant changes in IPAQ scores (*p* = 0.006, *v* = 0.263), but no significant changes in sitting times.

### 3.2 What predicts life satisfaction: regression analyses

Our regression analyses revealed complex relationships between physical activity patterns and life satisfaction, with some findings challenging conventional assumptions. The 2018 data on activity patterns among young Hungarian adults reveals interesting relationships between various types of physical activity and life satisfaction. Travel habits show a remarkable impact. Those who use a vehicle for travel just once a week report higher satisfaction compared to daily vehicle users. This pattern could reflect various lifestyle factors, such as having flexible work arrangements that reduce commuting needs, living closer to daily destinations, or having access to alternative transportation options. Cycling emerges as a strongly positive factor. Interestingly, almost any frequency of cycling (except daily) is associated with higher life satisfaction compared to daily cycling. This could indicate that while cycling is beneficial, having it as a daily necessity might be less enjoyable (Table 2).

**Table 2:** Relationships between demographic variables, physical activity and life satisfaction among young Hungarian adults.

Predictor	2018	2024
Demographic variables		
Birth year	OR: 1.142 (1.021–1.276); *p* = 0.021	OR: 1.168 (1.042–1.309); *p* = 0.008
Height	NS	OR: 1.048 (1.018–1.079); *p* = 0.002
Weight	NS	OR: 0.970 (0.955–0.985); *p* < 0.001
Education level (compared to doctoral degree)	OR range: 50.56–812.94; *p* < 0.05	NS
Settlement type (compared to municipality)	NS	County town: OR: 1.848 (1.037–3.291); *p* = 0.037
		City: OR: 1.719 (1.072–2.756); *p* = 0.025
Marital status (unmarried compared to relationship)	NS	OR: 0.650 (0.449–0.942); *p* = 0.024
Number of people in household (compared to 7)	NS	2 people: OR: 10.207 (1.509–69.075); *p* = 0.018
		6 people: OR: 8.776 (1.164–66.129); *p* = 0.036
Economic status (compared to other inactive)	NS	OR range: 11.168–40.244; *p* < 0.05
Number of employees at company (11–24 vs. not working)	OR: 0.216 (0.048–0.968); *p* = 0.044	NS
Accommodation type	Traditional farmhouse: OR: 0.114 (0.014–0.917); *p* = 0.040	Multi-apartment non-green: OR: 0.284 (0.087–0.931); *p* = 0.038
Net monthly family income (€1,351–€1,486 vs. no answer)	NS	OR: 4.011 (1.545–10.412); *p* = 0.004
Monthly costs	€243–€257: OR: 0.139 (0.032–0.606); *p* = 0.010	€257–€405: OR: 0.327 (0.130–0.822); *p* = 0.017
Illness in last 3 months	OR: 0.394 (0.209–0.743); *p* = 0.004	NS
Illness in last year	OR: 0.422 (0.193–0.924); *p* = 0.031	OR: 0.508 (0.321–0.804); *p* = 0.004
Average cost of medicines per month (<€2.70 vs. > €13.51)	NS	OR: 0.522 (0.292–0.933); *p* = 0.028
Activities		
Travel by vehicle (1 vs. 7 days per week)	OR: 5.425 (1.201–24.517); *p* = 0.028	NS
Cycling from A to B (compared to 7 days per week)	OR range: 16.642–43.130; *p* < 0.05	NS
Hard work in garden/house (compared to 7 days)	0 days: OR: 4.402 (1.019–19.024); *p* = 0.047	OR range: 0.066–0.181; *p* < 0.05
		2 days: OR: 7.463 (1.498–37.164); *p* = 0.014
Sitting on weekdays (compared to 7 days)	OR range: 0.075–0.349; *p* < 0.05	NS
Sitting on weekend (0 vs. 2 days)	OR: 6.056 (1.546–23.716); *p* = 0.010	NS
Hard physical work at work (4 vs. 7 days per week)	NS	OR: 0.364 (0.137–0.969); *p* = 0.043
Walk from A to B (0 vs. 7 days per week)	NS	OR: 2.537 (1.124–5.726); *p* = 0.025
Light work in garden/house (2 vs. 7 days)	NS	OR: 3.658 (1.275–10.496); *p* = 0.016
Very hard exercise in free time (7 vs. 0 or 1 days)	NS	0 days: OR: 0.375 (0.179–0.786); *p* = 0.010
		1 day: OR: 0.375 (0.168–0.837); *p* = 0.017
IPAQ, MET, and derived variables		
MET distribution (Work vs. Leisure)	OR: 0.391 (0.216–0.708); *p* = 0.002	NS
Relationship status (In vs. Not in relationship)	OR: 0.588 (0.367–0.942); *p* = 0.027	OR: 0.674 (0.495–0.918); *p* = 0.012
Work type (Standing vs. Physical)	NS	OR: 0.441 (0.218–0.892); *p* = 0.023

OR, Odds Ratio; NS, Not Significant (*p* > 0.05); CI, Confidence Interval.

Non-significant values (NS) were indicated clearly for comparison. Significant results are bolded with appropriate OR values and *p*-values.

A particularly noteworthy finding concerns household physical activity. Engaging in hard work in the garden or house occasionally (0 or 2 days a week) is associated with higher satisfaction compared to doing it daily. This pattern likely reflects the difference between chosen and obligatory physical activity - occasional household work may represent voluntary engagement, while daily hard work might represent necessary chores that could diminish satisfaction.

Sitting patterns during weekdays show a clear trend: less sitting is generally associated with higher satisfaction. Those who sit less during weekdays report higher satisfaction levels compared to those who sit every day. This aligns with general health advice about the benefits of reducing sedentary time. Of particular importance is our finding that the overall level of physical activity (categorized as low, moderate, or high) did not show a significant relationship with life satisfaction. This unexpected result suggests that the context and type of physical activity may be more important than the total amount.

Respondents who reported minimal sitting time on weekends showed higher satisfaction. This could reflect engagement in more active leisure pursuits or participation in enjoyable activities that limit sedentary behavior.

Overall, these findings suggest that in 2018, young Hungarian adults who maintained a balance of physical activity - including cycling, some home-based physical work, and limited sitting time - tended to report higher life satisfaction. The data emphasizes the importance of regular movement and the potential benefits of an active lifestyle, both in daily routines and leisure time.

The 2024 data on activity patterns among young Hungarian adults reveals some interesting relationships between various types of physical activity and life satisfaction.

The 2024 data reveals several significant shifts in the relationship between physical activity and life satisfaction, potentially reflecting post-pandemic changes in lifestyle patterns. Regarding work-related activities, those who engage in hard physical work at their job for 4 days a week report lower satisfaction compared to those who perform such work at their job daily. This finding contradicts some previous research suggesting that less physical labor correlates with higher satisfaction, indicating that the relationship between work-related physical activity and well-being may be more complex than previously thought. This might suggest that a full work week of hard physical labor, while potentially demanding, could be associated with a sense of accomplishment or financial stability that contributes to life satisfaction.

Interestingly, those who walk from point A to B less than daily report higher satisfaction than those who do so every day. This could potentially be related to lifestyle factors, such as having access to more convenient transportation options or working from home.

When it comes to household activities, the frequency of hard work in the garden or house reveals a discernible pattern. Those engaging in such work daily report higher satisfaction levels than those who do so less frequently, marking a noteworthy departure from the 2018 findings. On one hand, this outcome could reflect a growing sense of accomplishment or pride in one’s living space, particularly in light of shifting lifestyle priorities following the pandemic. On the other hand, it is also plausible that individuals who dislike such tasks simply choose not to perform them daily, thus contributing to the observed differences in satisfaction.

On the other hand, light work in the garden or house seems to have an optimal frequency. Those doing this type of work 2 days a week report higher satisfaction than those doing it daily. This suggests that some engagement in light household work is beneficial, but daily commitment might be less enjoyable.

A particularly significant finding, which has important implications for public health recommendations, concerns leisure activities, very hard exercise in free time shows an interesting pattern. Those who never engage in very hard exercise or do so only once a week report lower satisfaction compared to those doing it daily. This aligns with research on the mental health benefits of regular intense physical activity. However, this relationship between high-intensity exercise and well-being appears more complex than previous literature suggests, particularly when considering the broader context of our findings about overall physical activity levels.

Another notable finding that challenges conventional wisdom concerns the link between relationship status and life satisfaction. Our data consistently shows lower life satisfaction among those in relationships compared to single individuals, even when controlling for other variables. While this unexpected pattern could stem from the multifaceted strains of modern partnerships—such as work-life balance, shared physical activity routines, and competing lifestyle preferences—it may also be tied to financial constraints that make leaving a less fulfilling relationship more difficult.

These findings, while sometimes counterintuitive, should be interpreted within the specific context of young Hungarian adults and the post-pandemic period. The results highlight the complex interplay between physical activity, life satisfaction, and various social and environmental factors unique to this population.

## 4 Discussion

### 4.1 IPAQ and SBQ related implications

The results reveal notable trends and changes in physical activity levels and sitting times among young adults in Hungary between 2018 and 2024. The most striking changes occurred in IPAQ scores, indicating a shift in activity levels across several demographic groups. The COVID-19 pandemic likely played a significant role in these changes, as lockdowns and restrictions could have influenced physical activity habits and increased sedentary behavior. However, in contrast to the international trends, unlike previous research, which confirmed increased sitting time during COVID-19 due to the rise in home-office work and screen-based leisure activities (computer, phone, tablet) (55–58), the results of this study show an increase in higher levels of physical activity and a decrease in sitting time post-pandemic. The observed trends in physical activity, sedentary behavior, and life satisfaction reflect both real societal changes and the methodological differences in sampling between 2018 and 2024.

The significant increase in high IPAQ scores among males suggests an overall increase in physical activity levels. The reduction in weekday sitting times further supports this finding, indicating that males may have adopted more active lifestyles. Several international studies have also confirmed higher activity levels among men (59–61). For females, the significant increase in high IPAQ scores suggests a similar trend, although the changes in sitting times were not statistically significant. This gender difference may reflect broader societal patterns in Hungary, where traditional gender roles and responsibilities can influence physical activity opportunities.

The geographic distribution of physical activity changes reveals important patterns specific to the Hungarian context. The significant changes in IPAQ scores and sitting times among respondents from county seats and cities suggest a shift toward more active lifestyles in these areas. This trend could be due to increased access to recreational facilities and outdoor spaces (62, 63), as well as changes in work and commuting patterns. Notably, the lack of significant changes in the capital city and municipalities highlights the unique challenges faced in different Hungarian settlement types, where infrastructure and lifestyle opportunities vary considerably.

One of our most compelling findings concerns the relationship between occupational physical activity and life satisfaction. The significant reduction in weekday sitting times among respondents with sitting activities indicates a positive shift toward more active lifestyles. However, the lack of significant changes among those with mixed, standing, and physical activities suggests that these groups may have already had higher baseline activity levels.

The finding that standing jobs are associated with lower life satisfaction compared to more physically active jobs warrants particular attention. This could be attributed to several factors unique to the Hungarian work environment: The static nature of prolonged standing may lead to physical discomfort and fatigue. Standing jobs often offer less autonomy and fewer opportunities for varied movement. These positions typically occur in service sectors with other challenging working conditions. These insights suggest potential workplace interventions such as: implementation of dynamic workstations or introduction of structured movement breaks or job rotation systems to vary physical demands.

The significant changes in IPAQ scores among individuals in relationships suggest that having a partner may positively influence physical activity levels. The lack of significant changes in sitting times, however, indicates that sedentary behavior may still be prevalent among this group. The counterintuitive finding that individuals in relationships report lower life satisfaction merits careful interpretation within the Hungarian young adult context: high expectations and social pressures on young couples or economic challenges of establishing households or competing demands between relationship maintenance and personal well-being.

The significant changes in IPAQ scores among respondents with secondary and university education highlight the role of education in promoting active lifestyles (64). The lack of significant changes in sitting times suggests that while education may influence overall activity levels, it may not be sufficient to reduce sedentary behavior. These findings emphasize the need for comprehensive health promotion strategies that address both physical activity and sitting time, particularly among those with lower educational attainment (65).

### 4.2 Life satisfaction-related conclusions

Physical activity as a means of increasing life satisfaction was confirmed by Maher et al. (66). They conducted a diary study of 150 adults aged 18–89 and found that physical activity showed a positive correlation with life satisfaction. They also demonstrated that on days when people were more physically active than usual, they experienced greater life satisfaction. Life satisfaction in the study was lower among young adults and the older adults, while it was higher among middle-aged individuals.

Our findings regarding intensity levels present some intriguing contradictions with existing literature. There are contradictory results regarding the intensity levels of physical activity and subjective well-being. An international study found that the number of days spent in moderate-intensity physical activity positively correlated with subjective well-being; however, time spent in high-intensity physical activity negatively correlated with subjective well-being (67). This apparent contradiction might be explained by considering the specific context of young Hungarian adults: Cultural attitudes toward exercise intensity; Availability and accessibility of different exercise options; the relationship between exercise intensity and socioeconomic status.

## 5 Conclusion

The analysis of IPAQ and SBQ data from 2018 and 2024 highlights significant trends in physical activity levels and sedentary behavior among young adults in Hungary. These findings reflect both societal changes and the profound influence of the COVID-19 pandemic on daily routines and lifestyle patterns. However, it is important to acknowledge that the 2018 data were derived from a stratified random sample, while the 2024 data relied on a convenience sample.

While there has been a notable increase in physical activity, particularly among certain demographic groups, persistent sedentary behavior remains a critical challenge, especially during weekends and within specific occupational and relationship contexts. These trends underscore the need for tailored, multi-level interventions that account for both behavioral and systemic factors.

Although this study’s findings alone may not generate stakeholder-specific interventions, they nevertheless align with widely recognized best practices in reducing sedentary behavior and promoting balanced physical activity. Employers, for instance, can encourage periodic movement breaks, develop ergonomic workstations, and integrate voluntary wellness programs, thereby supporting both health and productivity. Public health authorities might tailor outreach efforts to young adults by emphasizing the pleasure and well-being derived from activity, rather than merely focusing on quantitative exercise goals. Urban planners could explore enhancements to communal infrastructure, including better cycling lanes, pedestrian-friendly zones, and green spaces, all of which foster more active daily routines. Educators and policymakers, meanwhile, may integrate lessons on physical well-being and sedentary habits into school and university curricula, helping nurture a culture of consistent and enjoyable physical engagement. While these suggestions draw on a broader public health context rather than the study’s data alone, they illustrate potentially constructive paths for mitigating some of the patterns observed in our research.

The comparison between 2018 and 2024 reveals deeper societal patterns in the role of physical activity and its relationship with life satisfaction. While the emergence of work-related factors, such as standing jobs, as significant predictors of life satisfaction highlights the evolving nature of occupational physical activity, it is essential to interpret these findings cautiously due to methodological differences between the two datasets.

The nuanced findings regarding home-based activities and exercise intensity point to a broader insight: physical activity is not inherently beneficial unless it aligns with individual preferences, lifestyles, and levels of autonomy. Promoting “purposeful” physical activity—where individuals find meaning, enjoyment, and balance—may offer a more sustainable path to improving life satisfaction among young adults.

Ultimately, the study underscores the dynamic interplay between physical activity, sedentary behavior, and life satisfaction, emphasizing the importance of ongoing, context-sensitive assessments. To ensure sustained progress, we recommend the formation of multi-disciplinary working groups comprising policymakers, urban planners, employers, health professionals, and community leaders. Such collaborative efforts will facilitate integrated interventions tailored to the unique needs and challenges faced by young Hungarian adults, fostering healthier, more fulfilling lifestyles.

## 6 Limitations and future research

The International Physical Activity Questionnaire (IPAQ) and the Sedentary Behavior Questionnaire (SBQ) are widely used self-reported tools for measuring physical activity and sedentary behavior, but both questionnaires have several limitations when it comes to accurate data collection. One of the biggest issues with the IPAQ is the inaccuracy of self-reporting, as individuals tend to overestimate their level of physical activity, especially with regard to vigorous activities. Additionally, the reliability of responses may be affected by memory biases, as individuals may have difficulty accurately recalling the activities they performed over the past week. The IPAQ also does not account for cultural differences, which may influence the interpretation of responses and the comparability of results. The subjective assessment of activity intensity, as well as the exclusion of non-recreational physical activities, further limits the usefulness of the questionnaire. Similar limitations affect the SBQ, which does not clearly differentiate among diverse sedentary pursuits—such as television viewing, computer use, or time spent on mobile devices—and therefore may obscure important variations in overall sedentary behavior. Individuals often struggle to accurately estimate the amount of time they spend in sedentary activities, especially when multiple activities are involved. Moreover, the questionnaire does not consider environmental factors that influence sedentary behavior, such as whether activities occur at home, at work, or in other settings. Additionally, the questionnaire does not assess how frequently sedentary periods are interrupted by short bouts of physical activity, which could be important for health. Since the SBQ also relies on subjective self-assessment, the results may be skewed and may not reflect the actual situation.

The study employs a cross-sectional design, comparing two independent samples collected at different time points. This approach does not allow for tracking changes in individual behaviors over time and cannot establish causality.

The period between 2018 and 2024 encompasses the COVID-19 pandemic, which significantly altered daily routines and behaviors. The observed changes in physical activity and sedentary behavior may reflect the pandemic’s short-term impact rather than long-term trends. Once pandemic-related restrictions were lifted, some behaviors may have reverted to pre-pandemic patterns, while others may have evolved. As a result, the findings may not be generalizable to other time periods or future scenarios.

The study focuses on a limited set of demographic variables—gender, settlement type, employment status, work type, relationship status, and educational level—to explore trends in physical activity and sedentary behavior. While these variables provide valuable insights, other potentially relevant factors, such as socioeconomic status, health conditions, regional disparities beyond settlement type, or access to recreational infrastructure, were not examined. Including these factors in future studies would enhance the depth and applicability of the findings.

The findings are specific to young adults aged 18–30 in Hungary. Cultural, environmental, and societal factors unique to Hungary may further limit the generalizability of the results to other populations or age groups. Comparative studies conducted across different countries, regions, and age groups would provide a broader understanding of these trends.

Future research should prioritize the use of longitudinal designs to track individual behavioral changes over time and establish causality. Incorporating objective measures of physical activity and sedentary behavior, such as wearable activity trackers, would enhance data accuracy and reduce self-report biases. Additionally, expanding the range of demographic and contextual variables and conducting cross-national comparative studies will allow for a more nuanced and comprehensive understanding of the factors influencing physical activity and sedentary behavior.

## Data Availability

The raw data supporting the conclusions of this article will be made available by the authors, without undue reservation.
